# Effects of *Bacillus subtilis* Natto Strains on Antiviral Responses in Resiquimod-Stimulated Human M1-Phenotype Macrophages

**DOI:** 10.3390/foods12020313

**Published:** 2023-01-09

**Authors:** Keisuke Fujii, Yuji Kubo, Tomotsugu Noguchi, Keisuke Tobita

**Affiliations:** Industrial Technology Innovation Center of Ibaraki Prefecture, Nagaoka 311-3195, Ibaraki, Japan

**Keywords:** *Bacillus subtilis* strains, macrophage, natto, resiquimod, ssRNA virus

## Abstract

*Bacillus subtilis* natto is used in the production of natto, a traditional fermented soy food, and has beneficial immunomodulatory effects in humans. Single-stranded RNA (ssRNA) viruses, including influenza and coronavirus, often cause global pandemics. We proposed a human cell culture model mimicking ssRNA viral infection and investigated the ability of *B. subtilis* natto to induce antiviral effects in the model. The gene expressions were analyzed using quantitative real-time reverse transcription PCR. M1-phenotype macrophages derived from THP-1 cells strongly express the Toll-like receptor 8 (76.2-hold), CD80 (64.2-hold), and CCR7 (45.7-hold) mRNA compared to M0 macrophages. One µg/mL of resiquimod (RSQ)-stimulation induced the expression of IRF3 (1.9-hold), CXCL10 (14.5-hold), IFNβ1 (3.5-hold), ISG20 (4.4-hold), and MxA (1.7-hold) mRNA in the M1-phenotype macrophages. Based on these results, the RSQ-stimulated M1-phenotype macrophages were used as a cell culture model mimicking ssRNA viral infection. Moreover, the *B. subtilis* natto XF36 strain induced the expression of genes associated with antiviral activities (IFNβ1, IFNλ1, ISG20, and RNase L) and anti-inflammatory activities (IL-10) in the cell culture model. Thus, it is suggested that the XF36 suppresses viral infections and excessive inflammation by inducing the expression of genes involved in antiviral and anti-inflammatory activities.

## 1. Introduction

Natto is a traditional Japanese fermented soybean food with a history of more than 1000 years [1]. Natto is made by fermenting soybeans steamed with *Bacillus subtilis* natto and is beneficial for health. *B. subtilis* natto is a gram-positive spore-forming bacterium known to play a role in health care as a probiotic [2,3]. Recently, it was reported that the spore cells of the *B. subtilis* natto strain isolated from natto modulate the immune system via Th1 cytokines, such as interleukin 12 (IL-12) in macrophage cells [4].

Macrophages that control the immune system are classified as M1- and M2-phenotype macrophages based on their different roles [5]. M1-phenotype macrophages (classically activated macrophages) are activated by infection with pathogens and are involved in killing pathogens by inducing proinflammatory cytokines [5]. CD86, iNOS, and TNFα gene expression are observed in M1-phenotype macrophages [5]. M2-phenotype macrophages (alternatively activated macrophages) are involved in tissue repair and allergic responses in Th2 differentiation [5]. In particular, promoting M1-phenotype macrophage polarization is essential in defense against viral infection [6].

Viruses are mainly composed of nucleic acids and protein membranes that enclose them. The most fundamental difference among viruses is their diversity in genome replication [7]. These are roughly classified into DNA and RNA viruses, according to the type of genome replication [7]. Single-stranded RNA (ssRNA) viruses, like the Ebola virus, influenza virus, severe acute respiratory syndrome coronavirus (SARS-CoV)-1, and SARS-CoV-2, have been responsible for pandemics [8].

Pattern recognition receptors (PRRs) recognize a variety of microbial components and play an essential role in the development of innate and adaptive immune responses [9]. Toll-like receptors (TLRs) recognize lipopolysaccharides, lipoprotein, flagellin, and nucleic acids, such as DNA and RNA, derived from pathogens [9]. Nucleic acid-recognizing TLRs (TLR3, TLR7, TLR8, and TLR9) are localized within the endosomal compartments [10]. Double-stranded RNA (dsRNA) or unmethylated CpG-DNA (CpG) motif directly bind to TLR3 or TLR9, respectively [10]. Human TLR7 or TLR8 recognizes viral ssRNA and synthetic oligoribonucleotides, such as imidazoquinoline, imiquimod (IMQ), or resiquimod (RSQ) [11]. The activation of TLR7 and TLR8 signals leads to the production of IL-1, IL-6, MIP-1, TNFα, and type 1 interferons (IFNs) [12]. The viral ssRNA that invade the body are recognized by TLR7 and TLR8 expressed in endosomes of M1-phenotype macrophages, and innate immune responses, such as proinflammatory reactions, are initiated [13,14].

Generally, viral infection models use virus mimetics agonists synthesized to target nucleic acid-recognizing TLRs. These models are safer than live or attenuated viruses and allow control of the proinflammatory response with dosage manipulation. The most common virus infection-like model uses polyinosinic-polycytidylic acid (Poly IC), a synthetic dsRNA, which thus stimulates TLR3 [15]. However, this model failed to replicate the stimulation from infection of ssRNA viruses such as SARS-CoV-1 and SARS-CoV-2 [16].

RSQ is known to elicit innate immune responses in the lungs of neonatal mice, similar to influenza viruses [17]. Moreover, intraperitoneal injection of RSQ in mice is useful in vivo model of ssRNA virus infection, which induces pro-inflammation, such as CXCL10 in the periphery and central nervous system, and virus infection-like illness [16]. This virus-free model using RSQ would provide safety to experimenters. However, based on animal welfare, it is desired to develop the cell model without using experimental animals.

The aim of this study is to propose a cell culture model to mimic ssRNA viral infection using RSQ-stimulated human M1-phenotype macrophage cells, and to investigate the ability of the *B. subtilis* natto strain to induce antiviral effects in the cell culture model.

## 2. Materials and Methods

### 2.1. Materials

Heat-inactivated fetal bovine serum (FBS) was sourced from HyClone Lab (Logan, UT, USA). IMQ and RSQ were purchased from InvivoGen (San Diego, CA, USA). CpG ODN 2006 (CpG) was purchased from Hycult Biotech Inc. (Wayne, PA, USA). Recombinant human IFNγ, IL-4, and IL-13 were purchased from R&D Systems, Inc. (Minneapolis, MN, USA). Poly IC and macrophage-activating lipopeptide-2 (MALP-2) were purchased from Novus International Inc. (St. Charles, MO, USA). *Escherichia coli* O111-derived lipopolysaccharide (LPS), phorbol 12-myristate 13-acetate (PMA), and 1% (*v*/*v*) penicillin and streptomycin solution were purchased from FUJIFILM Wako Pure Chemical Corp. (Osaka, Japan). The highest commercial analytical grade chemicals were used.

### 2.2. M1-Phenotype Macrophages Derived from THP-1 Cells

THP-1 cells (JCRB0112.1) are registered with the JCRB Cell Bank (Osaka, Japan). The cells were cultured in Roswell Park Memorial Institute (RPMI)-1640 media containing 10% (*v*/*v*) FBS, 2 mmol/L L-glutamine, and 1% (*v*/*v*) penicillin and streptomycin solution (RPMI-1640 basal medium). The cells were grown at 37 °C with 5% (*v*/*v*) CO_2_ and 95% (*v*/*v*) air (hereafter, the same conditions). M1-phenotype macrophages were prepared from THP-1 according to the reports of Genin et al., (2015) [18]. Briefly, 1 × 10^6^ cells/mL were suspended in RPMI-1640 basal medium containing 100 ng/mL of PMA and seeded in 3 mL each on six-well plates and cultured for 48 h. Fresh RPMI-1640 basal medium was added to the adhered cells, cultured for 24 h, and differentiated into macrophages (M0 macrophages). Differentiated macrophages were suspended in RPMI-1640 basal medium containing 100 ng/mL of LPS and 20 ng/mL of IFNγ and cultured for 48 h. The cells were suspended with RPMI-1640 basal medium and used in the experiment as M1-phenotype macrophages.

### 2.3. Quantitative Real-Time Reverse Transcription PCR (qRT-PCR)

The qRT-PCR was used to examine mRNA expression according to the reports of Tobita and Meguro (2022) [4]. TRIzol reagent (Invitrogen, Carlsbad, CA, USA) was used to extract total RNA from the cells. The cells were suspended in 1 mL TRIzol reagent, and 200 μL of chloroform was added, followed by centrifugation at 11,000 rpm for 15 min. The aqueous phases were transferred to a new tube, 500 μL of isopropanol was added, and centrifuged at 11,000 rpm for 15 min. After removing the supernatants, the pellets were washed with 1 mL of 75% (*v*/*v*) ethanol. The pellets were dissolved in RNase-free ultrapure water. The concentration of total RNA in each sample was measured as the A_260_/A_280_ ratio. The PrimeScript RT Reagent Kit with gDNA Eraser was used to accomplish the RT reaction (Takara Bio Inc., Shiga, Japan). TaqMan gene expression assays and TaqMan gene expression master mix were used for PCR (Thermo Fisher Scientific Inc., Waltham, MA, USA). The list of the TaqMan gene expression assays is shown in Table 1. The PCR reaction had 45 cycles of 95 °C for 1 s and 60 °C for 20 s. Gene expression was measured by relative quantitation (RQ). We used the comparative C_T_ (ΔΔC_T_) method to calculate the RQ of mRNA expression, using glyceraldehyde-3-phosphate dehydrogenase (GAPDH) mRNA as the endogenous control for mRNA expression. All the experimental data are presented as the mean expression index (n-fold change relative) to the control.

### 2.4. Stimulation of TLR Ligands in M1-Phenotype Macrophage Cell Cultures

The TLR2/TLR6 ligand (MALP-2), TLR3 ligand (Poly IC), TLR7 ligand (IMQ), TLR7/TLR8 ligand (RSQ), or TLR9 ligand (CpG ODN) were added in M1-phenotype macrophage cell cultures. The cells were cultured at 37 °C with 5% (*v*/*v*) CO_2_ and 95% (*v*/*v*) air for 3 h.

### 2.5. Bacterial Cultures

The *B. subtilis* natto strains (XF36, lst-1, Namegata-2-2, and Miyagi-4100) were used as a starter for the production of natto (fermented soybeans) and are stocked at the Industrial Technology Innovation Center of Ibaraki Prefecture (Ibaraki, Japan) [19,20,21,22]. *B. subtilis* NBRC3134 was sold from the National Institute of Technology and Evaluation (Kisarazu, Chiba). These strains were pre-cultivated overnight at 37 °C by shaking in Luria–Bertani medium (10 g tryptone, 5 g yeast extract, and 10 g NaCl in 1 L distilled water). The spore cells were obtained by inoculating them onto Schaeffer’s sporulation medium agar and cultivating them for 48 h at 37 °C. After confirming that the ratio of spore cells was typically over 99%, using phase-contrast microscopy, these cells were harvested. These cells were centrifugally washed thrice with sterile water, and lyophilized. The lyophilized bacteria were stored at −80 °C.

### 2.6. Stimulation of RSQ and B. subtilis Natto Strains in M1-Phenotype Macrophage Cell Cultures

**Figure 1:**
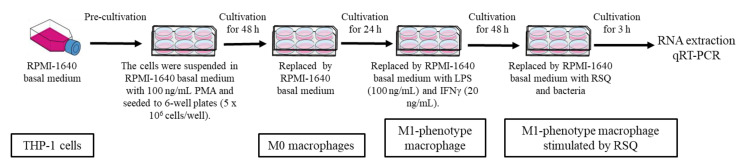
Schedule of stimulation with RSQ and bacteria in M1-phenotype macrophage cell cultures.

The schedule of stimulation with RSQ and bacteria in M1-phenotype macrophage cell cultures is shown in Figure 1. The lyophilized *B. subtilis* natto strains were suspended in 0.01 M phosphate-buffered saline (PBS; pH 7.2) and employed in the test as the bacterial suspension. M1-phenotype macrophage cell cultures were incubated with RSQ (1 µg/mL) and the bacterial suspension (10 μg/mL) at 37 °C with 5% (*v*/*v*) CO_2_ and 95% (*v*/*v*) air for 3 h.

### 2.7. Statistics

The data are presented as the mean and standard deviation (SD). Following one-way analysis of variance (ANOVA), the difference between different groups was evaluated using the Student’s *t*-tests of two independent samples, Tukey’s test, or Dunnett’s test for multiple comparison tests. Excel Statistics 2018 was used to analyze: The Social Survey Research Information Co., Ltd., Tokyo, Japan. The differences were statistically significant when the *p* values were less than 0.05.

## 3. Results

### 3.1. Macrophage Polarization toward M1 Phenotype

The mRNA relative expression values of PRRs and M1-phenotype macrophage markers are shown in Table 2. Compared to PMA-stimulated THP-1 cells (M0 macrophages), LPS and IFNγ-stimulated M0 macrophages upregulated the mRNA expression of TLR1 (1.6 ± 0.1), TLR2 (4.8 ± 0.5), TLR3 (4.9 ± 0.5), TLR8 (76.2 ± 9.0), TLR9 (1.5 ± 0.2), RIG-I (4.4 ± 0.7), MD2 (1.5 ± 0.2), myeloid differentiation factor 88 (MyD88) (2.2 ± 0.4), NFκB (3.2 ± 0.2), and NOD2 (5.8 ± 0.6) (*p* < 0.05). The relative expression values of CD40 (9.0 ± 1.1), CD80 (64.2 ± 23.2), iNOS (2.1 ± 0.6), and CCR7 (25.7 ± 16.1) in LPS and IFNγ-stimulated M0 macrophages were significantly elevated compared to those in M0 macrophages (*p* < 0.05). There was no difference in mRNA expression of TLR4 (1.0 ± 0.1), TLR6 (1.2 ± 0.3), NOD1 (1.2 ± 0.1), and IL-12β (2.0 ± 1.4) between these cells.

### 3.2. Immune Responsiveness of M1-Phenotype Macrophages after Stimulation of RSQ

**Figure 2:**
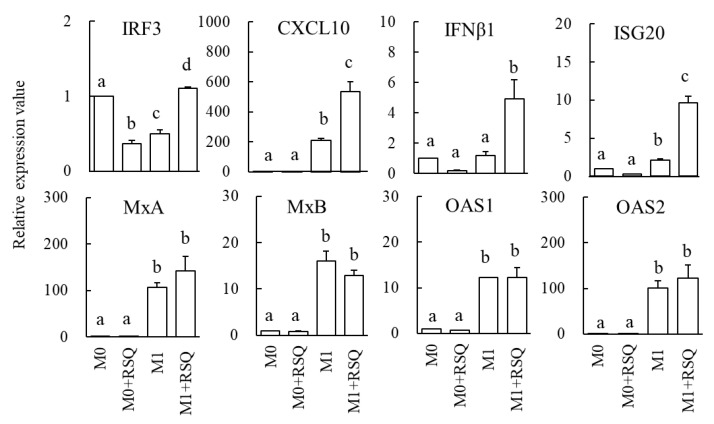
Effects of resiquimod (RSQ) on the expression of antivirus factor genes in M0 or M1-phenotype macrophages. M0 and M1-phenotype macrophages were cultivated in the presence of 1 μg/mL RSQ for 3 h. Quantitative real-time reverse transcription polymerase chain reaction assessed the relative expression values. The data are presented as the mean and SD (*n* = 3). The comparison among multiple groups was evaluated using Tukey’s test posttest following ANOVA. Different letters represent a statistically significant difference, *p* < 0.05.

Immune responsiveness of M0 and M1-phenotype macrophages after stimulation of RSQ is shown in Figure 2. Relative gene expression values when comparing M0 macrophages are shown below in the order of RSQ-stimulated M0 macrophages, M1-phenotype macrophages, and RSQ-stimulated M1-phenotype macrophages. IRF3; 0.4 ± 0.0, 0.5 ± 0.0, 1.1 ± 0.0. CXCL10; 0.5 ± 0.0, 210.0 ± 14.6, 533.6 ± 66.8. IFNβ1; 0.2 ± 0.0, 1.2 ± 0.2, 4.9 ± 1.3. ISG20; 0.3 ± 0.0, 2.1 ± 0.1, 9.7 ± 0.8. MxA; 0.5 ± 0.1, 106.1 ± 10.9, 142.6 ± 30.1. MxB; 0.5 ± 0.1, 106.1 ± 10.9, 142.6 ± 30.1. OAS1; 0.7 ± 0.0, 12.2 ± 0.0, 12.3 ± 2.0. OAS2; 0.7 ± 0.1, 100.8 ± 16.7, 123.2 ± 28.9. CXCL10 mRNA expression was higher in RSQ-stimulated and unstimulated M1-phenotype macrophages compared to M0 macrophages (*p* < 0.05). There was no difference in CXCL10 mRNA expression between unstimulated and RSQ-stimulated M0 macrophages. There was no difference in expression of IFNβ1, ISG20, myxovirus resistance (Mx) A, MxB, OAS1, and OAS2 mRNA between unstimulated and RSQ-stimulated M0 macrophages. Expression of IFNβ1 mRNA was significantly higher in RSQ-stimulated M1-phenotype macrophages compared to unstimulated M0 or M1-phenotype macrophages (*p* < 0.05). Expressions of ISG20, MxA, MxB, OAS1, and OAS2 mRNA were higher in unstimulated and RSQ-stimulated M1-phenotype macrophages than those in unstimulated and RSQ-stimulated M0 macrophages (*p* < 0.05). The immune responsiveness, such as increased IFNβ1 and ISG20 genes toward RSQ, was higher in M1-phenotype macrophages compared to M0 phenotype macrophages (*p* < 0.05).

**Figure 3:**
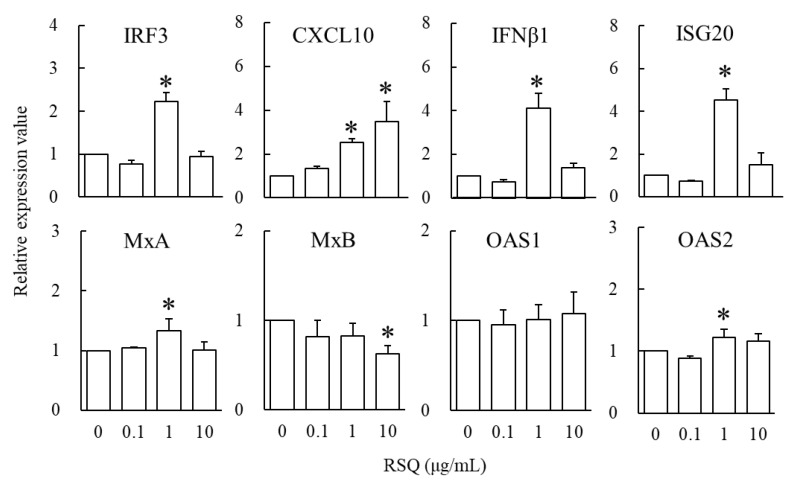
Effects of resiquimod (RSQ) on the expression of antiviral factor genes in M1-phenotype macrophages. M1-phenotype macrophages were cultivated in the presence of RSQ (0–10 μg/mL) for 3 h. Quantitative real-time reverse transcription polymerase chain reaction assessed the relative expression values. The data are presented as the mean and SD (*n* = 3). The comparison among multiple groups was evaluated using Dunnett’s posttest following ANOVA. * *p* < 0.05 (vs. 0 μg/mL RSQ).

Furthermore, the effect of RSQ concentration on the immune responsiveness in M1-phenotype macrophages was investigated. The expression of IRF3, CXCL10, IFNβ1, ISG20, MxA, MxB, OAS1, and OAS2 mRNA are shown in Figure 3. Relative gene expression values when comparing the cells without RSQ (0 µg/mL) are shown below in the order of 0.1, 1, and 10 µg/mL RSQ. IRF3; 0.8 ± 0.1, 2.2 ± 0.2, 0.9 ± 0.1. CXCL10; 1.3 ± 0.1, 2.5 ± 0.1, 3.5 ± 0.9. IFNβ1; 0.7 ± 0.1, 4.1 ± 0.7, 1.4 ± 0.2. ISG20; 0.7 ± 0.0, 4.5 ± 0.5, 1.5 ± 0.5. MxA; 1.0 ± 0.0, 1.3 ± 0.2, 1.0 ± 0.1. MxB; 0.8 ± 0.2, 0.8 ± 0.1, 0.6 ± 0.1. OAS1; 1.0 ± 0.2, 1.0 ± 0.2, 1.1 ± 0.2. OAS2; 0.9 ± 0.0, 1.2 ± 0.1, 1.2 ± 0.1. The expression of IRF3, CXCL10, IFNβ1, ISG20, MxA, and OAS2 mRNA were significantly higher in the cells with 1 µg/mL RSQ than in those without RSQ (*p* < 0.05).

### 3.3. Comparison of Immune Responsiveness of M1-Phenotype Macrophages after Stimulation of RSQ and Other Synthetic TLR Ligands

**Figure 4:**
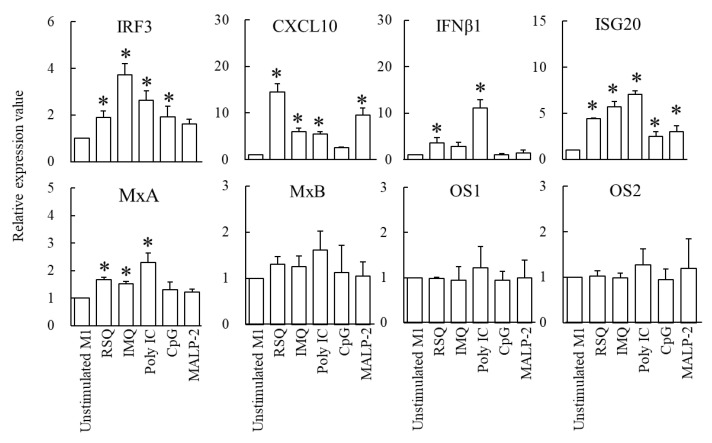
Effects of toll-like receptors (TLRs) ligands on the expression of antiviral genes in M1-phenotype macrophages. M1-phenotype macrophages were cultivated in the presence of phosphate-buffered saline (unstimulated M1) or TLR ligands (10 μg/mL resiquimod, 10 μg/mL imiquimod, 1 μg/mL polyinosinic-polycytidylic acid, 20 nmol/mL CpG, or 100 ng/mL macrophage-activating lipopeptide-2) for 3 h. Quantitative real-time reverse transcription polymerase chain reaction assessed the relative expression values. The data are presented as the mean and SD (*n* = 3). The comparison among multiple groups was evaluated using Dunnett’s posttest following ANOVA. * *p* < 0.05 (vs. unstimulated M1).

The immune responsiveness of M1-phenotype macrophages toward stimulating synthetic TLR ligands containing RSQ is shown in Figure 4. Relative gene expression values when comparing unstimulated M1-phenotype macrophages are shown below in the order of stimulation of RSQ, IMQ, Poly IC, CpG, and MALP-2. IRF3; 1.9 ± 0.3, 3.7 ± 0.5, 2.6 ± 0.4, 1.9 ± 0.5, 1.6 ± 0.2. CXCL10; 14.5 ± 1.9, 6.0 ± 0.7, 5.5 ± 0.5, 2.5 ± 0.2, 9.6 ± 1.4. IFNβ1; 3.5 ± 1.2, 2.8 ± 1.0, 11.1 ± 1.7, 1.0 ± 0.2, 1.5 ± 0.6. ISG20; 4.4 ± 0.1, 5.7 ± 0.6, 7.1 ± 0.3, 2.5 ± 0.5, 3.0 ± 0.7. MxA; 1.7 ± 0.1, 1.5 ± 0.1, 2.3 ± 0.3, 1.3 ± 0.3, 1.2 ± 0.1. MxB; 1.3 ± 0.2, 1.3 ± 0.2, 1.6 ± 0.4, 1.1 ± 0.6, 1.1 ± 0.3. OAS1; 1.0 ± 0.0, 0.9 ± 0.3, 1.2 ± 0.5, 0.9 ± 0.2, 1.0 ± 0.4. OAS2; 1.0 ± 0.1, 1.0 ± 0.1, 1.3 ± 0.3, 1.0 ± 0.2, 1.2 ± 0.7. Expression of CXCL10 mRNA was significantly elevated by stimulation of RSQ, IMQ, Poly IC, or MALP-2 compared to unstimulated M1-phenotype macrophages (*p* < 0.05). Significantly, the expression of CXCL10 mRNA in RSQ-stimulated M1-phenotype macrophages was 14.5-fold higher compared to unstimulated M1-phenotype macrophages. Expression of IFNβ1 mRNA was induced by RSQ or Poly IC (*p* < 0.05). Expression of ISG20 mRNA was significantly higher in M1-phenotype macrophages stimulated with the provided ligands (RSQ, IMQ, Poly IC, CpG, or MALP-2) compared to unstimulated M1-phenotype macrophages (*p* < 0.05). Expression of MxA mRNA was significantly higher in M1-phenotype macrophages stimulated with the provided ligands (RSQ, IMQ, and Poly IC) compared to unstimulated M1-phenotype macrophages (*p* < 0.05).

### 3.4. Effects of B. subtilis Natto Strains on the Gene Expression of IFNs, ISGs, or Cytokines in the Cell Culture Model Mimicking ssRNA Viral Infection

**Figure 5:**
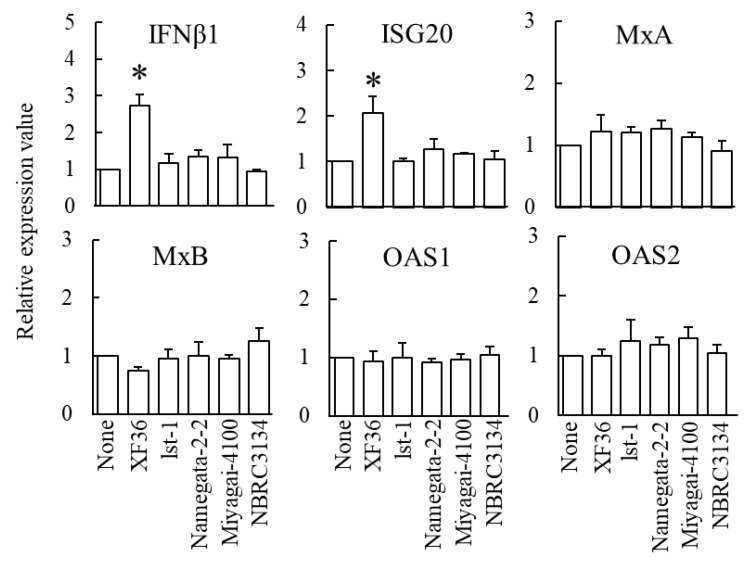
Effects of *B. subtilis* natto strains on the expression of antivirus factor genes in the cell culture model mimicking single-stranded RNA viral infection. M1-phenotype macrophages were cultivated with 1 µg/mL resiquimod (RSQ) only (none), 1 µg/mL RSQ, and 10 µg/mL *B. subtilis* natto strain (XF36, lst-1, Namegata-2-2, or Miyagi-4100), or 1 µg/mL RSQ, and 10 μg/mL *B. subtilis* NBRC3134 for 3 h. Quantitative real-time reverse transcription polymerase chain reaction assessed the relative expression values. The data are presented as the mean and SD (*n* = 3). The comparison among multiple groups was evaluated using Dunnett’s posttest following ANOVA. * *p* < 0.05 (vs. none).

It was investigated that the effects of *B. subtilis* natto strains on the gene expression of IFNs, ISGs, or cytokines in RSQ-stimulated M1-phenotype macrophages. The expressions of IFNβ1, ISG20, MxA, MxB, OAS1, and OAS2 mRNA in the cells are shown in Figure 5. Relative gene expression values when comparing RSQ-stimulated M1-phenotype macrophages are shown below in the order of stimulation of XF36, lst-1, Namegata-2-2, Miyagi-4100, and NBRC3134. IFNβ1; 2.7 ± 0.3, 1.2 ± 0.3, 1.3 ± 0.2, 1.3 ± 0.4, 0.9 ± 0.1. ISG20; 2.1 ± 0.4, 1.0 ± 0.1, 1.3 ± 0.2, 1.2 ± 0.0, 1.0 ± 0.2. MxA; 1.2 ± 0.3, 1.2 ± 0.1, 1.3 ± 0.1, 1.1 ± 0.1, 0.9 ± 0.2. MxB; 0.7 ± 0.1, 1.0 ± 0.1, 1.0 ± 0.2, 1.0 ± 0.1, 1.3 ± 0.2. OAS1; 0.9 ± 0.2, 1.0 ± 0.2, 0.9 ± 0.1, 1.0 ± 0.1, 1.0 ± 0.1. OAS2; 1.0 ± 0.1, 1.2 ± 0.4, 1.2 ± 0.1, 1.3 ± 0.2, 1.0 ± 0.1. IFNβ1 and ISG20 mRNA expression was significantly higher in the cells than in XF36 compared to the cells without bacteria (*p* < 0.05).

**Figure 6:**
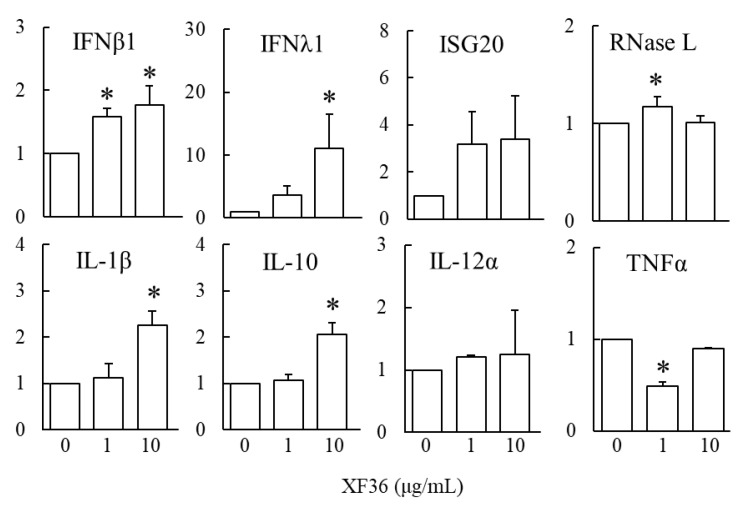
Effects of *B. subtilis* natto XF36 strain on the gene expression of interferons, interferon-stimulated genes, and cytokines in the cell culture model mimicking single-stranded RNA viral infection. M1-phenotype macrophages were cultivated with 1 µg/mL resiquimod (RSQ) and XF36 (0–10 µg/mL) for 3 h. Quantitative real-time reverse transcription polymerase chain reaction assessed the relative expression values. The data are presented as the mean and SD (*n* = 3). The comparison among multiple groups was evaluated using Dunnett’s posttest following ANOVA. * *p* < 0.05 (vs. 0 µg/mL XF36).

It was investigated that the effects of XF36 concentration on the gene expression of IFNs, ISGs, or cytokines in RSQ-stimulated M1-phenotype macrophages. The expressions of IFNβ1, IFNλ1, ISG20, RNase L, IL-1β, IL-10, IL-12α, and TNFα mRNA in the cells are shown in Figure 6. Relative gene expression values when comparing RSQ-stimulated M1-phenotype macrophages (0 µg/mL XF36) are shown below in the order of stimulation of 1 and 10 µg/mL XF36. IFNβ1; 1.6 ± 0.1, 1.8 ± 0.3. IFNλ1; 3.7 ± 1.4, 11.1 ± 5.5. ISG20; 3.2 ± 1.4, 3.4 ± 1.8. RNase L; 1.2 ± 0.1, 1.0 ± 0.1. IL-1β; 1.1 ± 0.3, 2.3 ± 0.3. IL-10; 1.1 ± 0.1, 2.1 ± 0.3. IL-12α; 1.2 ± 0.0, 1.3 ± 0.7. TNFα; 0.5 ± 0.0, 0.9 ± 0.0. The expression of IFNβ1 and RNase L mRNA was significantly higher in the cells with 1 μg/mL XF36 compared to the cells without XF36 (*p* < 0.05). The expression of IFNβ1, IFNλ1, IL-1β, and IL-10 mRNA was significantly higher in the cells with 10 μg/mL XF36 compared to the cells without XF36 (*p* < 0.05). Significantly, the expression of IFNβ1, IFNλ1, IL-1β, and IL-10 mRNA tended to increase depending on the concentration of XF36. The expression of TNFα mRNA was significantly lower in the cells with XF36 compared to the cells without XF36 (*p* < 0.05).

## 4. Discussion

PRRs containing TLRs play important roles in the development of innate and adaptive immunities and are strongly expressed in the M1-phenotype macrophages [23]. CCR7, IL-12, CD80, and iNOS are known markers of M1-phenotype macrophages [24]. The expressions of many PRRs and markers of M1-phenotype macrophages in LPS and IFNγ-stimulated M0 macrophages were increased compared to those in M0 macrophages (Table 2). These results suggest that LPS and IFNγ-stimulated macrophages derived from THP-1 cells function as M1-phenotype macrophages. Genin et al., (2015) [18] observed that M0 macrophages derived from THP-1 incubated with LPS and IFNγ expressed several M1-phenotype macrophage markers containing TNFα, IL-1β, IL-6, CXCL10, CD80, and HLA-DR. The present study found that M1-phenotype macrophages strongly expressed several PRRs in addition to those reported by Genin et al., (2015) [18]. In particular, it is interesting that the TLR8 expression is remarkably high in the cells.

M1-phenotype macrophages play an essential role in viral infection by producing inflammatory mediators [13,14]. Inflammatory mediators have been implicated in viral infections and the induction of disease behavior. Increased expression of specific chemokines has been reported in animal models and humans infected with ssRNA viruses [25]. CXCL10 was identified as a significant chemokine that is an attractant for monocytes, macrophages, dendritic cells, NK cells, and T cells [26]. In ex vivo human lung tissue explants, SASR-CoV-1 and SARS-CoV-2 inoculation strongly enhanced CXCL10 secretion in both cases [27]. Acute hepatitis A caused by hepatitis A virus infection is associated with the production of chemokines such as CXCL10, which is directly activated by IRF3 [28]. The SASR-CoV-1, SARS-CoV-2, and hepatitis A viruses are classified as ssRNA viruses [27,28]. Therefore, an increase in CXCL10 characterizes many ssRNA virus infections. In this study, expression of CXCL10 mRNA expression was higher in RSQ-stimulated and unstimulated M1-phenotype macrophages compared to M0 macrophages (Figure 2). Thus, it is suggested that RSQ-stimulation strongly induces CXCL10 mRNA expression in M1-phenotype macrophages but not M0 macrophages. Anfray et al., (2021) [29] reported that M1-phenotype macrophages differentiated from human monocytes produced CXCL10 compared to M0 macrophages. These findings support the results in this study.

The host’s response to viral infection causes initial activation of the PRRs, primarily by viral DNA and RNA. Signaling pathways activated by PRRs lead to the production of proinflammatory cytokines, recruitment of immune cells, expression of type I IFNs (such as IFNα and IFNβ) and type III IFNs (such as IFNλ1, IFNλ2, IFNλ3, and IFNλ4), and induction of ISGs [30]. ISGs are classified into several hundred types and exhibit antiviral effects [31]. ISG20 is the RNase to be induced by the IFNs system and acts on viral RNA degradation and translational inhibition [31]. MxA and MxB, dynamin-like GTPase genes, protect mammals from a wide range of viral infections. MxA in humans and mice reduces infection with RNA and DNA viruses [32]. MxB exhibits resistance to HIV-1 [32]. OAS1 and OAS2 are mediators of the antiviral responses via activation of the RNA cleavage pathway and synthesize 2-5A to activate RNase L [31]. In this study, gene expression of IFNβ1 was found to be elevated by RSQ stimulation in M1 phenotype macrophages (Figure 2). Furthermore, the gene expressions of ISGs, including ISG20, MxA, MxB, OAS1, and OAS2, were higher in M1-phenotype macrophages than in M0 macrophages (Figure 2). These results indicate that the IFNβ1 and ISGs are strongly induced in M1-phenotype macrophages compared to M0 macrophages. In addition, the immune responsiveness, such as increased IFNβ1 and ISG20 genes toward RSQ, was higher in M1-phenotype macrophages compared to M0 phenotype macrophages. These findings support the high expression of TLR8 mRNA in M1-phenotype macrophages, as shown in Table 2. Moreover, we investigated the effect of RSQ concentration on the immune responsiveness in M1-phenotype macrophages. The expression of IRF3, CXCL10, IFNβ1, ISG20, MxA, and OAS2 mRNA were significantly higher in the cells with 1 µg/mL RSQ than in those without RSQ (Figure 3). These results suggest that adding 1 µg/mL RSQ is optimal for immune responsiveness in M1-phenotype macrophages. These findings will allow control of the proinflammatory response with dosage manipulation.

The viral ssRNA binds and activates TLR7/8, induces the downstream adapter protein MyD88, and phosphorylates Interleukin 1R-Associated Kinase 1 (IRAK). IRAK1 induces phosphorylated IRF3 and IRF7, which translocate to the nucleus to generate type I IFNs [33]. IFNβ or IFNλ1 promoters are primarily activated by IRF3 [34]. In this study, RSQ enhanced the expressions of IFNβ1 and IRF3 mRNA in M1-phenotype macrophages (Figure 3). Thus, it is suggested that RSQ induces the expression of IFNβ via the activation of IRF3, similarly to the viral ssRNA.

TLR2, TLR3, TLR7, TLR8, and TLR9 recognize viral components, such as glycoprotein, ssRNA, dsRNA, and unmethylated CpG nucleotides [35]. Several synthetic ligands that bind to these TLRs induce inflammation. RSQ operates as ligands of TLR7 and TLR8. Unlike RSQ, IMQ activates only the TLR7 signal but not the TLR8 signal [36]. Poly IC, a synthetic mimetic of viral dsRNA, is recognized by TLR3 and induces the characteristic inflammatory responses associated with a viral infection, such as production of mucus and inflammatory cytokines [15,37]. TLR9 recognizes unmethylated CpG ODN and strongly induces Th1 immune responses [10]. MALP-2 is recognized by dimers composed of TLR2 and TLR6 and induces the production of inflammatory cytokines [9,38]. In particular, poly IC-induced inflammation has been studied in various airway epithelial cells. Poly IC stimulation of airway epithelial cells has been shown to mimic the inflammatory response associated with viral infection [15]. A dose-dependent of poly IC increase was observed in secretion of IL-6, IL-8, and TNFα in human bronchial epithelial [15]. However, little is known about the effect of poly IC stimulation on CXCL10 expression in M1-phenotype macrophages. In this study, we compared the immune responsiveness of M1-phenotype macrophages after stimulation with RSQ and other synthetic TLR ligands. RSQ stimulation strongly promoted CXCL10 expression compared to other synthetic TLR ligands, including poly IC (Figure 4). Moreover, it was observed that the stimulation of RSQ strongly promoted not only the gene expressions of ISG20 and IFNβ1, but also the gene expression of CXCL10 in M1-phenotype macrophages (Figure 4). These results suggest that the stimulation of RSQ induces immune responses similar to ssRNA virus infection than stimulation with other TLR ligands. Yates et al., (2022) [16] proposed the mice intraperitoneally injected with RSQ as an animal model of ssRNA viral infection showing high expression of chemokines, such as CXCL10. In this study, we found that RSQ-stimulated M1-phenotype macrophages strongly expressed CXCL10. Thus, we propose that the RSQ-stimulated M1-phenotypic macrophage cell culture is a cell culture model mimicking ssRNA viral infection. This virus-free model using RSQ would provide safety to experimenters. The model could be used to investigate the potential antiviral effects of various food and pharmaceutical ingredients without the use of animals.

We investigated the effects of *B. subtilis* natto strains on the gene expression of IFNs, ISGs, or cytokines in the cell culture model mimicking ssRNA viral infection. IFNβ1 and ISG20 mRNA expression was significantly higher in the cell culture model than in XF36 compared to the cell culture model without bacteria (Figure 5). These results suggest that XF36 strongly induces the expression of IFNβ1 and ISG20 mRNA compared to other strains of *B. subtilis*. It was shown that the effects on gene expression differ depending on the strain of *B. subtilis* added. The Honcho Shokan, published in 1697, states that the consumption of natto alleviates intestinal infections [39]. Ishida et al., (2003) [40] reported that the immunostimulatory activities of probiotic bacteria, such as lactic acid bacteria, depend on the strain rather than the species. Mazanko et al., (2022) [41] reported that *B. subtilis* strains isolated from poultry microbiota had different effects on growth performance and immune system in broilers. Our results appear to support these findings.

IL-1β leads to IFN production and activation of IFN signaling and induces a potent innate immune response against viral infections [42]. RNase L, activated by type I IFNs, restricts viral replication via cleaving viral ssRNA and causes apoptosis of the infected cells [43]. Inflammatory cytokines, such as TNFα and IL-12, play significant roles in the body’s inflammatory response to SARS-CoV-2 infection [44]. IL-10, an anti-inflammatory cytokine, protects the host from tissue damage in excessive inflammatory responses [45]. In the cell culture model, we investigated the effects of XF36 concentration on the gene expression of IFNs (type I and type III IFNs), ISGs, and cytokines (proinflammatory and anti-inflammatory cytokines). In this study, the expression of IFNβ1, IFNλ1, RNase L, IL-1β, and IL-10 mRNA was significantly higher in the cell culture model with XF36 compared to the cell culture model without XF36 (Figure 6). These results suggest that XF36 promotes the expression of genes associated with antiviral and anti-inflammatory effects in the cell culture model.

Although the protective effect of *B. subtilis* natto against infectious diseases is not well known, several studies have been performed using the *B. subtilis* spore cells to protect against infection in mice and humans [46,47]. Respiratory syncytial virus (RSV), which infects the lower respiratory tract, causes respiratory disease. Hong et al., (2019) [48] showed that intranasal administration of *B. subtilis* spore cells to RSV-infected mice promoted the production of IFNβ and IL-12 to alveolar macrophages, induced antiviral activity, and protected against RSV-induced lung damage. However, these reports do not clarify antiviral factors, including ISGs. In the complex immune system, it may be difficult to explain the detailed antiviral effects in vivo. In this study, XF36 promoted the expressions of IFNβ1, IFNλ1, ISG20, and RNase L (Figure 5 and Figure 6). These results suggest that the XF36 promotes the expression of ISGs through the induction of expression of IFNs. Thus, we have elucidated the mechanism of antiviral effects by XF36 using the cell culture model mimicking ssRNA viral infection.

IL-10 production is one of the hallmarks in M2-phenotype macrophages [5]. IL-10 produced by M2-phenotype macrophages shifts the immune activation toward tissue repair [5]. In this study, the XF36 promoted the expression of IL-10 mRNA, but not inflammatory cytokines, such as IL-12α and TNFα (Figure 6). These results suggest that the XF36 promotes differentiation from M1-phenotype to M2-phenotype macrophages. *Lactobacillus johnsonii* N6.2 strain, one of the probiotics, released nano-sized vesicles (NVs) with different protein and lipid contents [49]. Furthermore, the NVs promoted the M2 phenotype by producing IL-10 in macrophages derived from THP-1 cells [49]. *L. murinus* alleviated intestinal ischemia by promoting the production of IL-10 from M2-phenotype macrophages via TLR2 signaling in mice [50]. Therefore, the cell component of XF36 may promote the differentiation of M2-phenotype macrophages.

It is not clear how the ingested XF36 acts in the digestive tract. Gut-associated lymphoid tissue (GALT) plays an important role in intestinal immune responses. Peyer’s patches are clusters of GALT concentrated in the terminal ileum and contain lymphocytes, dendritic cells, macrophages, and villous epithelium [51]. Lopez-Guerrero et al., (2010) [52] reported that antigen-presenting cells, such as dendritic cells, produced TNFα and IFNβ during rotavirus infection in Peyer’s patches, and contributed to the control of infection in the intestine. On the other hand, spore cells of *B. subtilis* isolated from natto can survive under acidic conditions, such as gastric acid, and reach the intestine alive [3]. In this study, spore cells of *B. subtilis* XF36 induced antiviral and anti-inflammatory effects in the cell culture model mimicking ssRNA viral infection. Thus, orally ingested XF36 spore cells are expected to reach the intestine and promote the antiviral and anti-inflammatory effects via stimulation of macrophages in the Peyer’s patches during viral infection.

## 5. Conclusions

Herein, we developed a human cell culture model that mimics the host responses to ssRNA viruses by stimulating TLR7/8 ligand RSQ in M1-phenotype macrophages derived from THP-1 cells. Using the cell culture model, we investigated the antiviral effects of *B. subtilis* natto strains on gene expression. The XF36 strain induced the gene expression of IFNs, ISGs, and anti-inflammatory cytokines in the cell culture model. Thus, the XF36 strain suppresses viral infection and excessive inflammation by inducing the gene expression of antiviral and anti-inflammatory factors. These findings support the new beneficial effects of *B. subtilis* natto. *B. subtilis* natto is characterized as a food material that alleviates viral infections. Therefore, it is hoped that clinical trials will be conducted in the future.

## Figures and Tables

**Table 1:** TaqMan gene expression assays used in this study.

Gene	Cat No	Gene	Cat No	Gene	Cat No
CCR7	Hs01013469_m1	IRF3	Hs01547283_m1	TLR1	Hs00413978_m1
CD40	Hs00386848_m1	ISG20	Hs00158122_m1	TLR2	Hs01872448_s1
CD80	Hs01045162_m1	MD2	Hs00209770_m1	TLR3	Hs00152933_m1
CXCL10	Hs00171042_m1	MxA	Hs00895608_m1	TLR4	Hs00152939_m1
GAPDH	Hs02786624_g1	MxB	Hs01550813_m1	TLR6	Hs01039989_s1
IFNβ1	Hs01077958_s1	MyD88	Hs01573837_g1	TLR7	Hs01933259_s1
IFNλ1	Hs00601677_g1	NFκB1	Hs00765730_m1	TLR8	Hs07292888_s1
IL-1β	Hs01555410_m1	NOD1	Hs01036720_m1	TLR9	Hs00370913_s1
IL-10	Hs00961622_m1	NOD2	Hs01550753_m1	TNFα	Hs00174128_m1
IL-12α	Hs01073447_m1	OAS2	Hs00942643_m1	RIG-I	Hs01061444_m1
IL-12β	Hs01011518_m1	OAS1	Hs00973635_m1	RNase L	Hs00221692_m1
iNOS	Hs01075529_m1				

**Table 2:** The mRNA expressions of PRRs and M1-phenotype macrophage markers.

Gene	Relative Expression Value	Gene	Relative Expression Value	Gene	Relative Expression Value
TLR1	1.6 ± 0.1 *	TLR9	1.5 ± 0.2 *	CD40	9.0 ± 1.1 *
TLR2	4.8 ± 0.5 *	RIG-I	4.4 ± 0.7 *	CD80	64.2 ± 23.2 *
TLR3	4.9 ± 0.5 *	MD2	1.5 ± 0.2 *	iNOS	2.1 ± 0.6 *
TLR4	1.0 ± 0.1	MyD88	2.2 ± 0.4 *	IL-12β	2.0 ± 1.4
TLR6	1.2 ± 0.3	NFκB1	3.2 ± 0.2 *	CCR7	45.7 ± 16.1 *
TLR7	0.5 ± 0.0 *	NOD1	1.2 ± 0.1		
TLR8	76.2 ± 9.0 *	NOD2	5.8 ± 0.6 *		

The relative value of mRNA expressions was determined by qRT-PCR. The expression value of each mRNA was normalized to GAPDH mRNA expression. All experimental data are expressed as the mean of relative expression value (n-fold change relative) to the normalized expression value in M0 macrophages with SD (*n* = 3). The difference between M0 and M1-phenotype macrophages was evaluated using the Student’s *t*-tests. * *p* < 0.05.

## Data Availability

The data are contained within the article.
